# Chronic Physical Stress Does Not Interact with Epstein-Barr Virus (EBV)-Encoded Dutpase to Alter the Sickness Response

**DOI:** 10.4236/jbbs.2015.511049

**Published:** 2015-10-27

**Authors:** Taryn G. Aubrecht, Zachary M. Weil, Bachir Abi Salloum, Maria Eugenia Ariza, Marshall Williams, Brenda Reader, Ronald Glaser, John Sheridan, Randy J. Nelson

**Affiliations:** 1 Departments of Neuroscience, The Ohio State University, Columbus, OH, USA; 2 Molecular Virology, Immunology & Medical Genetics, The Ohio State University, Columbus, OH, USA; 3 Institute of Behavioral Medicine Research, Wexner Medical Center, Comprehensive Cancer Center, The Ohio State University, Columbus, OH, USA; 4 Division of Biosciences, College of Dentistry, The Ohio State University, Columbus, OH, USA

**Keywords:** Epstein-Barr Virus, Swim Stress, Sickness Response

## Abstract

Most adult humans have been infected with Epstein-Barr virus (EBV), which is thought to contribute to the development of chronic fatigue syndrome. Stress is known to influence the immune system and can exacerbate the sickness response. Although a role for psychological stress in the sickness response, particularly in combination with EBV-encoded deoxyuridine triphosphate nucleotidohydrolase (dUTPase) has been established, and the role of physical stressors in these interactions remains unspecified. In this study, we seek to determine the interaction of chronic physical (swim) stress and EBV-encoded dUTPase injection. We hypothesize that a chronic physical stressor will exacerbate the sickness response following EBV-encoded dUTPase injection. To test this hypothesis mice receive daily injections of EBV-encoded dUTPase or vehicle and are subjected to 15 min of swim stress each day for 14 days or left unmanipulated. On the final evening of injections mice undergo behavioral testing. EBV-encoded dUTPase injection alone produces some sickness behaviors. The physical swimming stress does not alter the sickness response.

## 1. Introduction

Chronic fatigue syndrome is characterized by extreme fatigue, muscle pain, swollen lymph nodes, loss of memory, sore throat, stress, and depression [1] [2]. Epstein-Barr virus (EBV) is one potential cause of chronic fatigue syndrome [1] [3]. The sickness response, often including fever, is conserved across many mammalian species and supports recovery from illness [4] [5]. However, stress can exacerbate sickness responses and impede recovery.

Acute psychological stress in particular exacerbates immune responses. For example, healthy medical students decrease cellular immunity on the last exam day compared to a month prior to the exams [6]. Trigeminal ganglion cells taken from mice that experience restraint stress exhibit reduced capability to produce interferon-gamma in response to reactivation of herpes simplex virus type 1 [7]. In contrast, chronic stressors tend to impair adaptive immune responses. For instance, chronic restraint stress suppresses cell mediated immunity in rats [8]. However, the behavioral syndrome associated with acute inflammation (*i.e.* sickness behavior) is modulated by stress via at least two distinct pathways. First, there is the immunomodulatory effect of stress responses and there are also direct behavioral changes mediated by stress (e.g. stress induced depressive symptoms that may resemble sickness behaviors). In mice, both 28 days of chronic unpredictable stress and predatory stress impairs the response to lipopolysaccharide [9]. Similarly, EBV-encoded deoxyuridine triphosphate nucleotidohydrolase (dUTPase) induces a sickness response in mice that is exacerbated by chronic restraint stress [10] [11].

In contrast to psychological stress, physical stress is less widely studied. In moth larvae, non-lethal physical stress primes the immune system to increase the immune response following exposure to a microbial pathogen [12]. Rats exposed to a chronic physical stressor (electrical foot shock) acutely suppress the blastic response of their splenic lymphocytes but chronic effects on splenic lymphocytes are only found in psychologically stressed rats, underlining the importance of examining both types of stressor effects on immune function [13]. Similarly, we previously demonstrate that the chronic restraint, a psychological stressor, impairs delayed-type hypersensitivity responses and leads to trafficking of leukocytes out of the peripheral blood. However, repeated forced swimming does not alter these parameters [14] even though it significantly increases circulating corticosterone concentrations. The effects of EBV-encoded dUTPase in combination with a chronic physical stressor remain unspecified.

Given the effects of a psychological stressor (restraint) exacerbating the response to EBV-encoded dUTPase injections, we aim to determine if a chronic physical stressor produces similar outcomes. Physical stressors do not elicit the same physiological response as psychological stressors. Therefore, investigating the interaction of swimming (physical) stress with sickness behavior induced by EBV-encoded dUTPase is an important follow up to our results with psychological stress. We hypothesize that chronic swimming stress exacerbates sickness behavior elicited by EBV-encoded dUTPase.

## 2. Materials and Methods

### 2.1. Subcloning and Purification of EBV-Encoded dUTPase

The subcloning of the EBV (BLLF3 pET3A was kindly provided by Dr. Peter Sommer (Institut fur Mikrobiologic und Hygiene, Abteilung Virolgie)was conducted by PCR amplification using the forward (5’-CCGGTTA-AGCTTGGATCCATGGAGGCC TGTC-3’) and reverse (5’-GCGAATTCTCATTGACCCGACGA TCC-3’) primer sets (125 pmol of each), DNA (140 ng), high fidelity PCR supermix (Invitrogen, Gary Island, NY, USA) and the following PCR conditions: denaturation at 94°C for 3’ (1 cycle) followed by 35 cycles of 94°C for 30 seconds (sec.), 50°C for 30 sec., 72°C for 1’ and one cycle at 72°C for 20’. The PCR product was purified using the QIAquick gel extraction kit (QIAGEN) and cloned into the protein expression vector pTrcHis Topo (Invitrogen, Gary Island, NY, USA). Twenty individual clones were isolated following transformation of *E. coli* Top 10 competent cells, DNA was then purified using the QIAPrep Spin Miniprep kit (QIAGEN, Valencia, CA, USA), screened by PCR for the presence of specific dUTPase genes and the sequence verified by DNA sequencing analysis. The pTrcHis dUTconstructs, containing the EBV-encoded dUTPase gene in the correct orientation and in frame, were used to transform *E. coli* BL21 (DE3) plyS competent cells for purification of recombinant proteins as described below.

The recombinant EBV-encoded dUTPase protein was purified using HisPur™ Spin columns (3 ml resin bed) as described by the manufacturer (Pierce, Rockford, IL, USA). Briefly, BL21(DE3) plyS containing a specific PTrcHisDUT construct was grown in LB medium containing chloramphenicol (25 μg/ml) and ampicillin (100 μg/ml) at 37°C for 2.5 h IPTG (1 mM final concentration) was added and the culture was incubated an additional 2 h at 37°C. Bacteria were collected from 1 - 2 liters of medium by low speed centrifugation and the bacterial pellet was resuspended in 50 ml of extraction buffer (50 mM sodium phosphate, 300 mM NaCl and 10 mM imidazole, pH 7.4). Bacteria were lysed by ultrasonication. The resulting homogenate was centrifuged (15,000 × g, 30 min at 4°C), and the supernatant was applied to a HisPur™ spin column, which was equilibrated in extraction buffer. The column was washed three times with two-resin bed volumes of extraction buffer and the EBV-encoded dUTPase protein was eluted by washing the column four times with one resin-bed volume of 50 mM sodium phosphate, 300 mM NaCl and 150 mM imidazole, pH 7.4. Fractions were assayed for the EBV-encoded dUTPase activity as described previously [15] and for protein using the Coomassie Brilliant Blue dye-binding assay (Bio-Rad Laboratories, Hercules, CA, USA) using bovine serum albumin as the standard. A unit of dUTPase activity was defined as the amount of enzyme required to convert 1 nM of dUTP to dUMP and pyrophosphate per min at 37°C under the assay conditions. Purity of EBV-encoded dUTPase was determined by SDS-PAGE as described previously [15]. Proteins were visualized using EZBlue™ protein gel stain as described by the manufacturer (Sigma Aldrich, St. Louis, MO). The EBV-encoded dUTPase protein preparations were tested as described previously [15] and were free of detectable levels of LPS, peptidoglycan (SLP-HS), DNA, or RNA.

### 2.2. Animals

Forty, six week old male CD-1 mice were purchased from Charles River, Inc. (Wilmington, MA, USA). Mice were group-housed, five per cage, in propylene cages (33 cm × 18 cm × 14 cm) at an ambient temperature of 22°C ± 2°C, relative humidity of 50% ± 10%. Animals were given *ad libitum* access to Harlan Teklad 8640 food (Madison, WI, USA) and filtered tap water. Mice were placed in a 12:12 light dark cycle and allowed to acclimate to the facility for at least one week.

One week after arrival mice had radiotelemetric transmitters (Mini Mitter, Respironics, Bend, OR, USA) implanted in the peritoneal cavity under isoflurane anesthesia. Mice were then moved to single-housing and were allowed to recover for one week. Home cages were placed on TR-4000 receiver boards connected to DP-24 Data Ports (Mini Mitter), that continuously collected temperature and activity data in 30 min bins. Activity represents beam breaks caused by motion and is therefore an overall index of locomotor activity and is not directly relatable to velocity or distance. These 30 min bins of activity and temperature data were extracted from the Vital View (Mini Mitter, Respironics, Bend, OR, USA) program at the conclusion of the experiment. Following one week of recovery, mice were injected in the right hindlimb muscle daily between 8:30 and 9:00 hours with 10 μg EBV-encoded dUTPase in saline or an equal volume of 0.9% saline. Mice were injected for 14 consecutive days.

Body weight was measured at the initiation and conclusion of the study. Food intake was measured daily starting two days before injections between 8:30 and 9:00 hours. Additionally, mice were given a 1:1 solution of water and sweetened condensed milk with 400 μg/mL of ferrous sulfate added daily for 3 h/day at the onset of the dark phase 18:00 hours [16]. Food and water were removed while mice had access to the sweetened condensed milk. On day 14 when mice were behaviorally tested they did not receive sweetened condensed milk.

### 2.3. Physical Stress

Starting the first day of inoculation, mice were moved to a separate room and underwent a 15 min forced swim trial in ~17 cm of 21°C ± 2°C water. The forced swim trials occurred at random times during the light phase (6:00 - 18:00 h) for the remainder of injections. No swim mice remained in the home cages. The combination of swim stress and injection make a total of four experimental groups (*n* = 10/group, **Table 1**).

### 2.4. Open Field

Starting at the onset of the dark phase (18:00 h) on the 14th day mice were behaviorally tested under red light. The open field test is used to characterize anxiety in a novel environment and locomotor activity. Central tendency is the primary measure for anxiety, defined as the proportion of time spent in the center of the open field. Locomotor activity was measured separately as total crosses between the center and periphery during testing. Mice were allowed to acclimate to the room for 20 min before testing. Mice were placed in a 40 cm × 40 cm clear acrylic chamber with the outside covered in cardboard to prevent mice from seeing the room. The center was defined as the central 28 cm × 28 cm. At the end of testing, mice were placed in a clean cage. Mice were recorded for 10 min during the open field and tapes were assessed by a condition blind observer using Observer software (Noldus Corp. Leesburg, VA) for time in the center versus periphery and number of crosses between the two areas.

### 2.5. Elevated Plus Maze

Five minutes after completion of the open field mice were tested on the elevated plus maze. The elevated plus maze measures anxiety-like behavior by examining time spent in the open arms versus time in the closed arms. Mice were placed in the center of the elevated plus maze. Mice were allowed to freely explore for 5 min and were recorded on video. Video was assessed by a condition blind observer using Observer software (Noldus Corp. Leesburg, VA) for time spent in the open arms.

### 2.6. Tail Suspension Test

Five minutes following the elevated plus maze mice were tested for depressive-like behavior using the tail suspension test, where more immobility equates to increased depressive-like behavior [17]. Mice were suspended by the distal part of their tail ~ 92 cm above the ground with electrical tape. Mice were recorded for 10 min and tapes were assessed by a condition blind observer using Observer software (Noldus Corp. Leesburg, VA, USA) for time immobile and time active.

### 2.7. Statistical Analysis

Main effects of restraint (no restraint versus restraint) and injection type (saline versus EBV-encoded dUTPase) and interactions between the two variables were assessed. Changes in body temperature for day 1 and changes averaged over all fourteen days were assessed with repeated measures ANOVAs. Activity on day 1 and averaged over the first seven, and fourteen days were assessed with repeated measures ANOVA. A univariate ANOVA was conducted on total food intake, total milk intake, change in body mass, percent of time in the center of the open field, and time in the open arm of the elevated plus maze. Due to unequal variance time frozen in the tail suspension test was log transformed to perform the univariate ANOVA. Statistics were performed using SPSS 19 for Windows (IBM, Armonk, New York, USA). Mean differences were considered statistically significant when *p* < 0.05. Significant differences were followed up with least significant differences post hoc tests.

## 3. Results

### 3.1. Body Temperature

**Figure 1:**
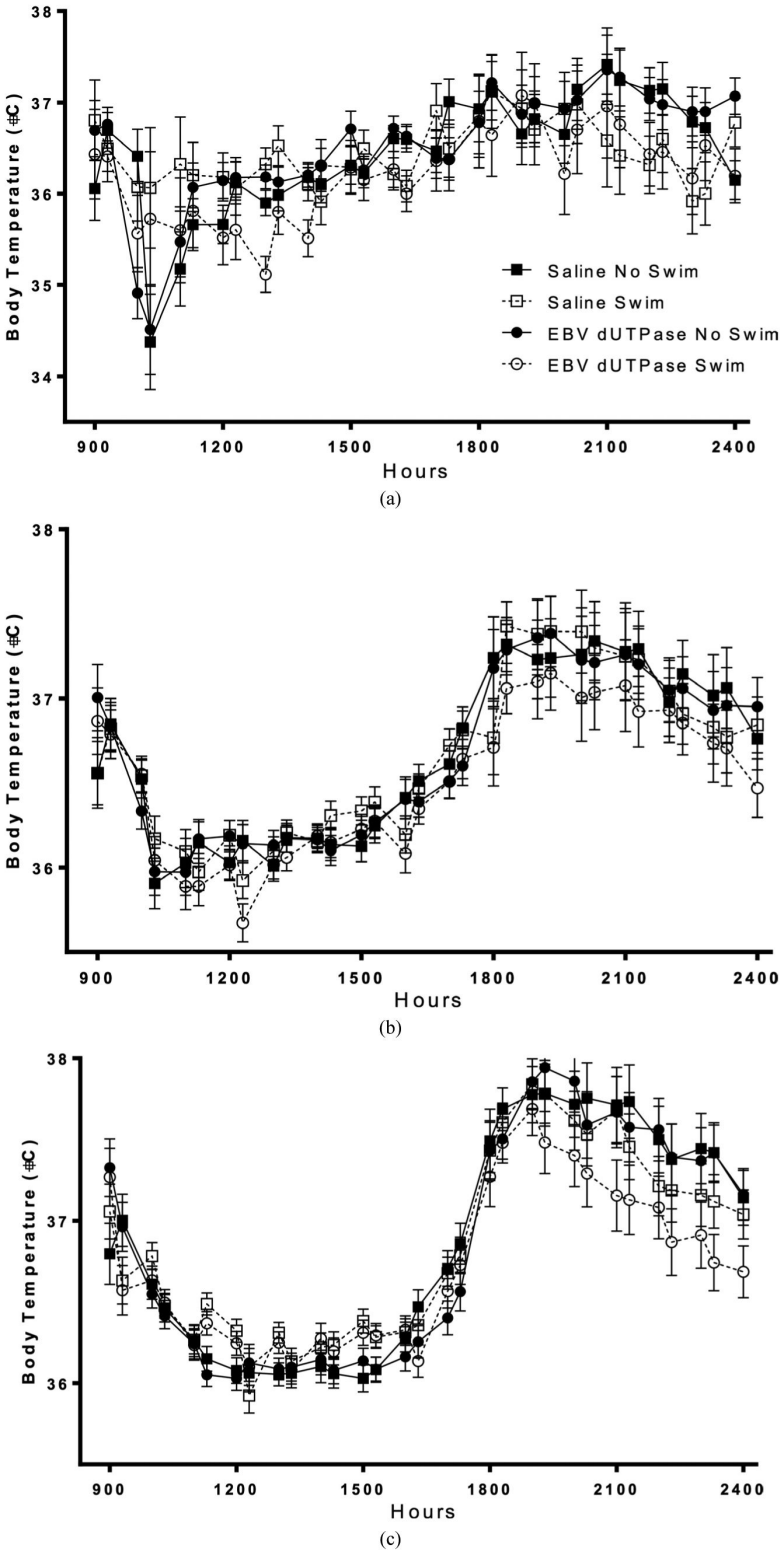

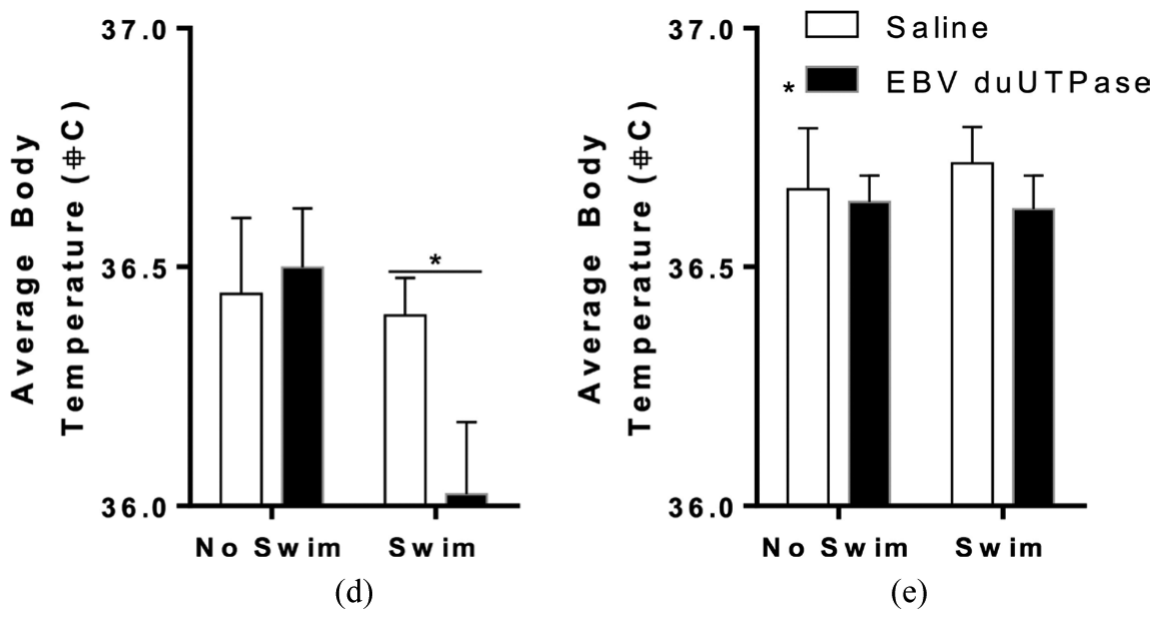
On day one, body temperature (±SEM) was reduced in mice that swam compared to mice that did not swim (a), significance marked on average body temperature graph for day 1 (d). Body temperature averaged from days 1 - 7 was not altered by swimming of EBV dUTPase injections (b). EBV dUTPase injections decreased body temperature compared to saline injections and swimming decreased body temperature compared to mice that did not swim averaged over all 14 days ((c), (e)). Each graph represents average body temperature (±SEM), across multiple days (for (b) and (c)), in the hours following injection. Asterisks (*) indicates significant main effect differences in repeated measures ANOVA, *p* < 0.05.

Body temperature on day one increased during the active phase (lights off) compared to inactive phase (lights on) (*F*_42,924_ = 8.68, *p* < 0.01, **Figure 1(a)**). On day one, body temperature was reduced (*F*_42,924_ = 2.11, *p* < 0.01, **Figure 1(a)**, **Figure 1(d)**) in mice that swam compared to mice that did not swim. Body temperature was not affected by EBV-encoded dUTPase (*p* > 0.05, **Figure 1(a)**) on day 1. Body temperature was not altered by EBV-encoded dUTPase or swimming averaged over the first seven days (*p* > 0.05, **Figure 1(b)**). Body temperature increased during the active phase (lights off) compared to inactive phase (lights on) (*F*_42,924_ = 49.6, *p* < 0.01, **Figure 1(b)**) averaged over the first 7 days. There was no interaction of EBV-encoded dUTPase and swimming on body temperature on any of the 14 days (*p* > 0.05, **Figure 1(c)**). EBV-encoded dUTPase injections decreased body temperature compared to saline injections (*F*_42,1344_ = 1.49, *p* < 0.05, **Figure 1(c)**, **Figure 1(e)**) averaged over all 14 days. Swimming decreased body temperature compared to mice that did not swim (*F*_42,1344_ = 1.49, *p* < 0.05, **Figure 1(c)**, **Figure 1(e)**) averaged over all 14 days. Body temperature increased during the active phase (lights off) compared to inactive phase (lights on) (*F*_42,924_ = 95.2, *p* < 0.01, **Figure 1(c)**) averaged over all 14 days.

### 3.2. Activity

**Figure 2:**
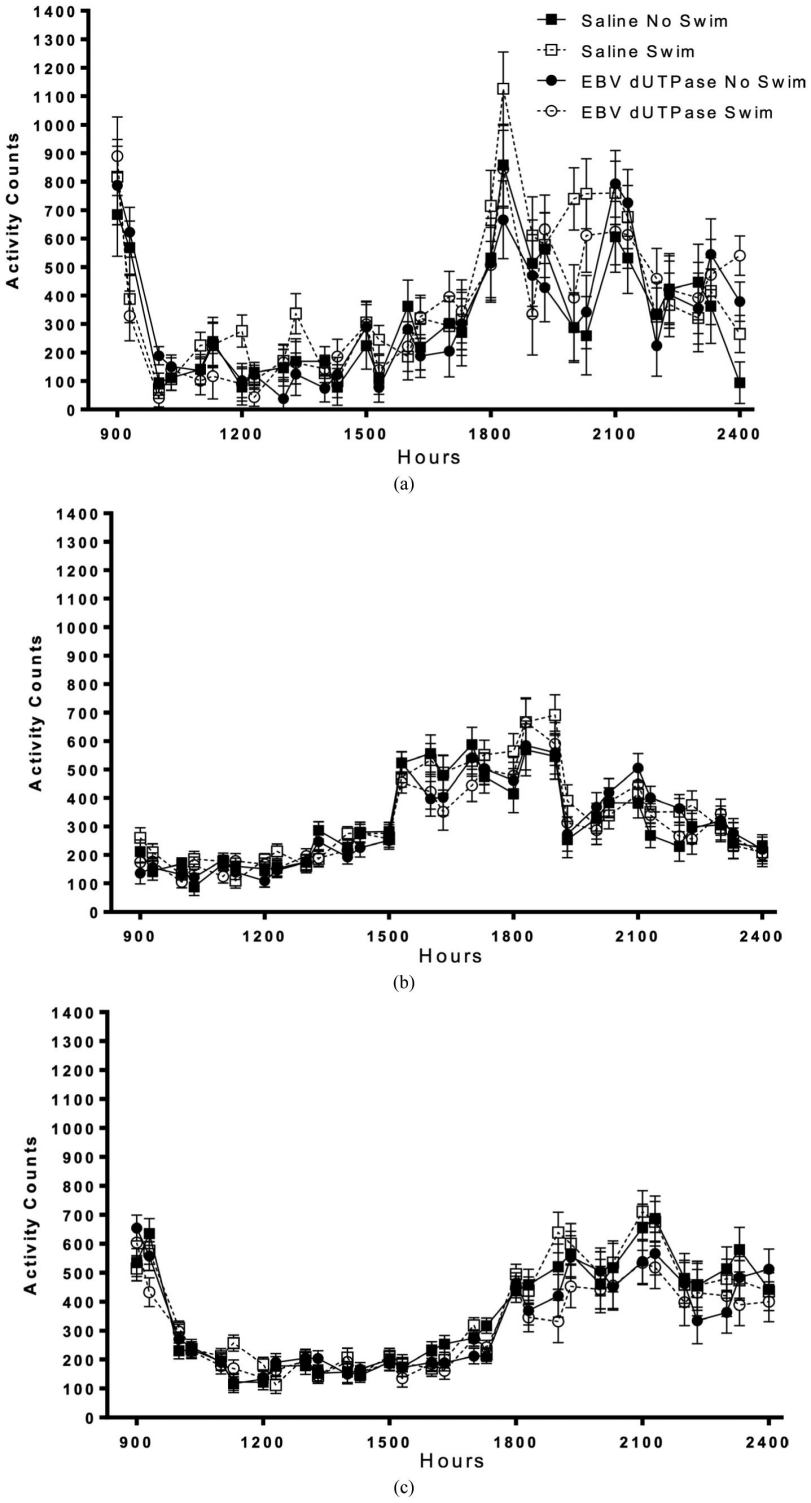

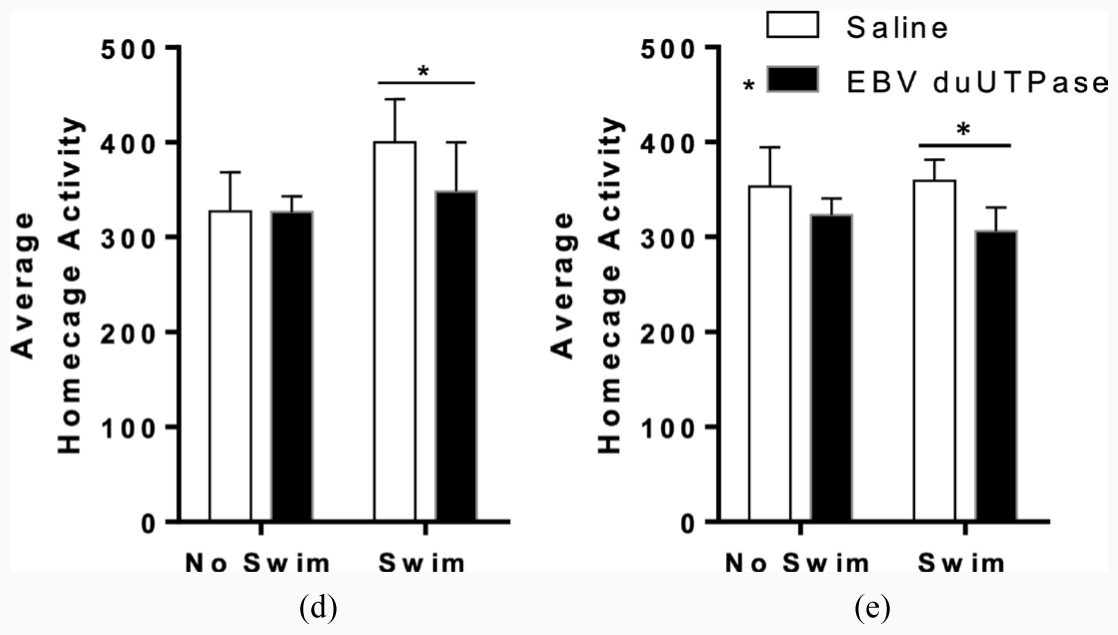
On day one home cage activity (±SEM) was increased in mice that swam ((a), (d)). Home cage activity averaged from days 1 - 7 was not altered by swimming of EBV dUTPase injections (b). EBV dUTPase injections decreased activity levels compared to saline injections averaged over all 14 days ((c), (e)). Each graph represents average activity (±SEM), across multiple days (for (b) and (c)), in the hours following injection. Asterisks (*) indicates significant main effect differences in repeated measures ANOVA, *p* < 0.05.

Activity increased during the active phase (lights off) compared to inactive phase (lights on) (*F*_42,1344_ = 17.7, *p* < 0.01, **Figure 2(a)**) on day one. On day one activity was reduced (*F*_42,1344_ = 1.47, *p* < 0.05, **Figure 2(a)**, **Figure 2(d)**) in mice that did not swim compared to mice that did swim. Activity was not affected by EBV-encoded dUTPase (*p* > 0.05, **Figure 2(a)**) on day 1. Activity levels were not altered by EBV-encoded dUTPase or swimming averaged over the first seven days (*p* > 0.05, **Figure 2(b)**). Activity increased during the active phase (lights off) compared to inactive phase (lights on) (*F*_42,1344_ = 38.3, *p* < 0.01, **Figure 2(b)**) averaged over the first 7 days. There was no interaction of EBV-encoded dUTPase and swimming on body temperature on any of the 14 days (*p* > 0.05, **Figure 2(c)**). EBV-encoded dUTPase injections decreased activity levels compared to saline injections (*F*_42,1344_ = 1.42, *p* < 0.05, **Figure 2(c)**, **Figure 2(e)**) averaged over all 14 days. Swimming did not alter activity levels averaged over all 14 days (*p* > 0.05, **Figure 2(c)**) compared to mice that did not swim. Activity increased during the active phase (lights off) compared to inactive phase (lights on) (*F*_42,1344_ = 35.9, *p* < 0.01, **Figure 2(c)**) averaged over all 14 days.

### 3.3. Food Intake

**Figure 3:**
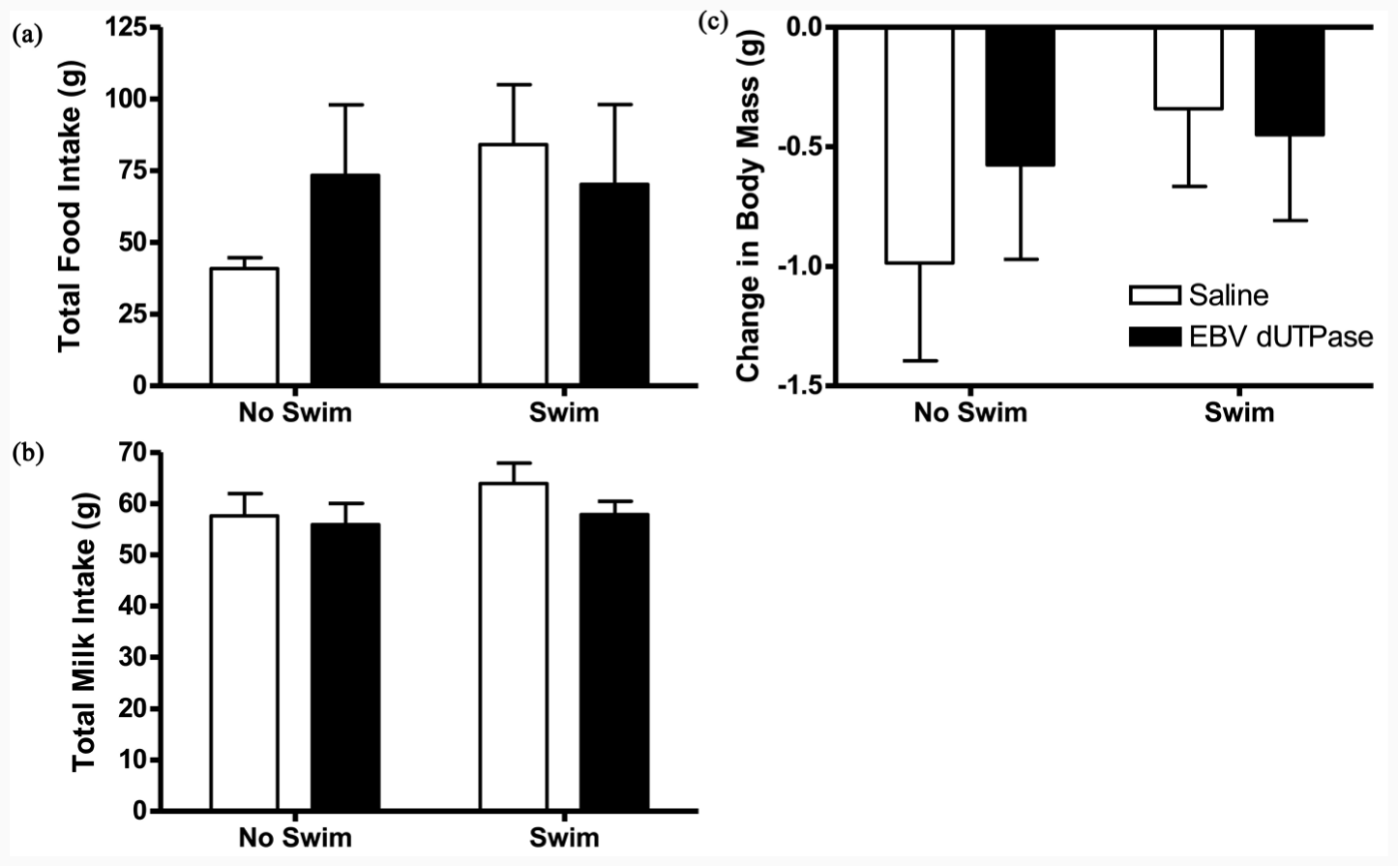
Data are presented as mean (±SEM) representing total food intake, milk intake (during three hour exposure daily), and change in body mass over all 14 days. Total food intake was not altered by EBV-encoded dUTPase injection (F_1,36_ = 0.19, *p* = 0.67, (a)) or swimming (F_1,36_ = 0.86, *p* = 0.36, (a)).Total milk intake was not altered by EBV-encoded dUTPase injection (F_1,36_ = 1.04, *p* = 0.31, (b)) or swimming (F_1,36_ = 1.16, *p* = 0.29, (b)). Change in body mass was not altered by EBV-encoded dUTPase injection (F_1,36_ = 0.16, *p* = 0.69, (c)) or swimming (F_1,36_ = 1.07, *p* = 0.31, (c)).

Total food intake was not altered by EBV-encoded dUTPase injection (F_1,36_ = 0.19, *p* = 0.67, **Figure 3(a)**) or swimming (F_1,36_ = 0.86, *p* = 0.36, **Figure 3(a)**).

### 3.4. Milk Intake

Total milk intake was not altered by EBV-encoded dUTPase injection (F_1,36_ = 1.04, *p* = 0.31, **Figure 3(b)**) or swimming (F_1,36_ = 1.16, *p* = 0.29, **Figure 3(b)**).

### 3.5. Body Mass

Change in body mass was not altered by EBV-encoded dUTPase injection (F_1,36_ = 0.16, *p* = 0.69, **Figure 3(c)**) or swimming (F_1,36_ = 1.07, *p* = 0.31, **Figure 3(c)**).

### 3.6. Open Field

**Figure 4:**
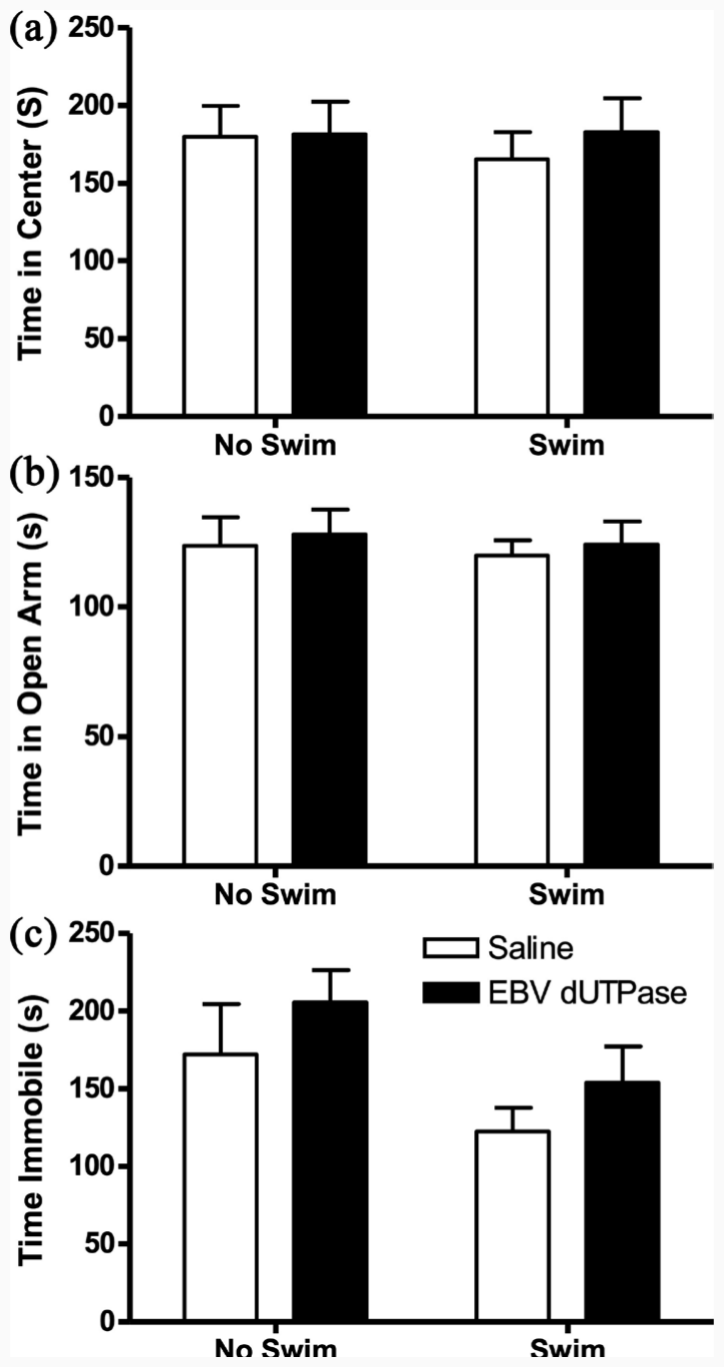
Anxiety- and depressive-like behaviors were similar among groups in the open field, elevated plus maze, and tail suspension test. Percent of time spent in the center of the open field on day 14 (a). Time spent on the open arm of the elevated plus maze on day 14 (b). Time spent immobile during the tail suspension test (c). All data are presented as mean (±SEM).

Anxiety-like behavior was not altered by EBV-encoded dUTPase injection (F_1,36_ = 0.11, *p* = 0.74, **Figure 4(a)**) or swimming (F_1,36_ = 0.21, *p* = 0.65, **Figure 4(a)**) assessed by time spent in the center of the open field.

### 3.7. Elevated Plus Maze

Anxiety-like behavior was not altered by EBV-encoded dUTPase injection (F_1,36_ = 0.23, *p* = 0.64, **Figure 4(b)**) or swimming (F_1,36_ = 0.17, *p* = 0.68, **Figure 4(b)**) assessed by time spent on the open arms.

### 3.8. Tail Suspension Test

Depressive-like behavior was not altered by EBV-encoded dUTPase injection (F_1,36_ = 1.34, *p* = 0.26, **Figure 4(c)**) or swimming (F_1,36_ = 3.64, *p* = 0.06, **Figure 4(c)**) assessed by time frozen during the tail suspension test.

## 4. Discussion

Approximately 95% of the human adult population has been infected with EBV [18]. Additionally, stress can exacerbate the sickness response and EBV-encoded dUTPase can elicit a sickness response independent of viral replication [10] [11]. The role of psychological versus physical stress in the sickness response, particularly in response to EBV-encoded dUTPase, remains unspecified. In this study, we hypothesized that a chronic physical stressor exacerbates the sickness response to EBV-encoded dUTPase. EBV-encoded dUTPase alone produced some sickness behaviors. Physical swimming stress did not produce a sickness response alone or in combination with EBV-encoded dUTPase injections.

Here we report that a chronic physical stressor, in this case a forced-swim procedure, does not modulate sickness responses to repeated treatment with the EBV-encoded dUTPase protein. These data are in contrast to our previous reports that chronic restraint stress exacerbates the sickness response induced by dUTPase. [10]. In rats, exposure to a single swim stress does not change central production of interleukin-1, a proinflammatory cytokine thought to play a role in alterations following psychological stress [19]. Proinflammatory cytokines are known to increase concurrent with sickness behaviors, for example following injection with lipopolysaccharide [20] [21]. Potentially, swim stress does not increase other proinflammatory cytokines and this may explain why there is no exacerbation of the sickness response. Similarly, voluntary exercise in young adult and aged mice did not attenuate inflammation or sickness behavior following lipopolysaccharide injection [22] [23]. Further, in a panel of different stressors forced swim failed to alter delayed-type hypersensitivity responses and leukocyte trafficking although these parameters were modulated by chronic restraint stress [14]. Additionally, the pattern of immediate early gene expression in the amygdala and neuroendocrine responses to forced swim and restraint differ markedly thus peripheral immune tissues are likely receiving very different physiological signals induced by the stress response [24]. Further, swim stress, because of the modulatory effect on body temperature may have other non-specific effects on immune physiology [25]. In any case, forced swim seems to be neither protective nor harmful in combination with an immune insult.

## 5. Conclusion

In conclusion, physical swim stress did not interact with EBV-encoded dUTPase injections. The effect of psychological restraint stress in combination with EBV injections was most pronounced with two weeks of concurrent treatment [10] [26]. However, even two weeks of chronic physical stress were not sufficient to exacerbate the sickness response following EBV-encoded dUTPase injections.

## Figures and Tables

**Table 1:** Experimental design including four groups, with 10 mice per group.

	Saline (*n*)	EBV-dUTPase (*n*)
No Swim	10	10
Swim	10	10

## Abbreviations

EBV: Epstein-Barr virus
dUTPase: deoxyuridine triphosphate nucleotidohydrolase
